# The Effect of Chitosan Incorporation on Physico-Mechanical and Biological Characteristics of a Calcium Silicate Filling Material

**DOI:** 10.3390/dj12040100

**Published:** 2024-04-10

**Authors:** Sumaya Abusrewil, J. Alun Scott, Saeed S. Alqahtani, Mark C. Butcher, Mohammed Tiba, Charchit Kumar, Daniel M. Mulvihill, Gordon Ramage, William McLean

**Affiliations:** 1 Glasgow Endodontology Group, Glasgow Dental School, School of Medicine, Dentistry and Nursing, College of Medical, Veterinary and Life Sciences, Glasgow G12 8QF, UK; 2224354a@student.gla.ac.uk (S.A.); 2195788a@student.gla.ac.uk (S.S.A.); 2538282t@student.gla.ac.uk (M.T.); william.mclean@glasgow.ac.uk (W.M.); 2 Oral Sciences Research Group, Glasgow Dental School, School of Medicine, Dentistry and Nursing, College of Medical, Veterinary and Life Sciences, University of Glasgow, 378 Sauchiehall Street, Glasgow G2 3JZ, UK; 2135158b@student.gla.ac.uk; 3 Materials and Manufacturing Research Group, James Watt, School of Engineering, University of Glasgow, Glasgow G12 8QQ, UK; charchit.kumar@glasgow.ac.uk (C.K.); daniel.mulvihill@glasgow.ac.uk (D.M.M.)

**Keywords:** biodentine, endodontics, dental material, tricalcium silicate cement, chitosan

## Abstract

Objectives: A tricalcium silicate-based cement, Biodentine™, has displayed antibiofilm activity when mixed with chitosan powder. This study aimed to assess the effect of chitosan incorporation on the physico-mechanical and biological properties of Biodentine™. Methods: In this study, medium molecular weight chitosan powder was incorporated into Biodentine™ in varying proportions (2.5 wt%, 5 wt%, 10 wt%, and 20 wt%). The setting time was determined using a Vicat apparatus, solubility was assessed by calculating weight variation after water immersion, radiopacity was evaluated and expressed in millimeters of aluminum, the compressive strength was evaluated using an Instron testing machine, and the microhardness was measured with a Vickers microhardness tester. In addition, surface topography of specimens was analyzed using scanning electron microscopy, and the effect of chitosan on the viability of human embryonic kidney (HEK 293) cells was measured by a colorimetric MTT assay. Results: Incorporation of 2.5 wt% and 5 wt% chitosan powder delivered an advantage by speeding up the setting time of Biodentine material. However, the incorporation of chitosan compromised all other material properties and the crystalline structure in a dose-dependent manner. The chitosan-modified material also showed significant decreases in the proliferation of the HEK 293 cells, signifying decreased biocompatibility. Significance: Chitosan incorporation into calcium silicate materials adversely affects the physical and biological properties of the material. Despite the increased antimicrobial activity of the modified material, the diminution in these properties is likely to reduce its clinical value.

## 1. Introduction 

Biodentine™ is a novel hydraulic calcium silicate material recognized for its high biocompatibility, bioactivity [1], outstanding sealing properties [2], and physicochemical properties superior to those of mineral trioxide aggregate (MTA) [3,4]. Such characteristics allow its use as a permanent dentine substitute in teeth. Its uses include vital pulp therapy, apexification, management of root resorption, retrograde root filling and perforation repairs [5,6]. 

In vitro testing of Biodentine, like other bioceramic materials, shows good antimicrobial activities. However, many of these experiments are conducted against planktonic microbes [7,8,9,10]. These studies do not really equate to the clinical reality where dental disease is caused by multispecies biofilms. When tested against multispecies biofilms, Biodentine is much less effective [11,12,13]. Although Biodentine has shown favorable clinical outcomes; for example, 94.4% at 2 years [14] and 91.7% at 3 years [15] when used in vital pulp therapy, there remains scope for improving the antimicrobial effects of such calcium silicate materials.

Studies have been performed with different types of additives blended into Biodentine in an attempt to obtain better material’s properties. Incorporation of 5% glass fiber powder into Biodentine was shown to significantly enhance the fracture resistance of obturated roots with the modified material when Biodentine was used as an intra-orifice barrier [16]. Aidaros and Niazy [17] assessed the pulp response to direct pulp capping with Biodentine incorporated with calcium phosphate nanoparticles (NPs). It was shown that the thickness of the dentine bridge formed by the new mixture was significantly greater than the one created by the unmodified Biodentine [17]. Silver NPs have also been tested with *Rosmarinus officinalis* L. extract (ROE) and Cefuroxime. The antibiofilm efficacy of Cefuroxime-ROE-AgNPs nano-antibiotics in Biodentine showed a significantly higher efficiency than Biodentine (unmodified), Cefuroxime, or ROE-AgNPs alone [18]. Chitosan is a modified natural carbohydrate polymer extracted from chitin [19], which is known to possess antimicrobial activity against a variety of bacterial strains and fungi [20]. The antimicrobial action of chitosan is not fully understood, but it could be attributed to electrostatic interactions that occur between the positively charged chitosan molecules and the negatively charged microbial components [21]. Samples which are completely dry are incapable of releasing their stored energy in chemical bonds to begin interactions [22]. Therefore, it is believed that the poor solubility of chitosan in water has limited its biological applications [23]. However, chitosan is soluble in a dilute aqueous acidic solution (pH < 6.5) [24], and its solubilization is generally carried out in aqueous acetic acid. Recently, powdered chitosan (medium molecular weight), in its insoluble form, has been shown to exhibit a dose-related response against multispecies biofilm when incorporated into Biodentine by 2.5 wt% and 5 wt% [11]. While this improvement in anti-microbial activity is beneficial, it is only one of the desired properties of the material. In order to allow the -modified Biodentine to function as designed clinically, it is essential that the other material properties of the bioceramic cement are maintained. In this study [25], it has been hypothesized that the addition of any substance, such as chitosan powder, is likely to affect the material properties of Biodentine. This potential for diminution of the physico-mechanical and biological characteristics has not been investigated in a previous study where the anti-microbial efficacy has been assessed [11]. Therefore, this study aims to investigate the effect of the incorporation of chitosan medium molecular weight on some of the physico-mechanical and biological properties of Biodentine, to assess any alterations that could have detrimental effects on its clinical efficacy. 

## 2. Materials and Methods 

**Figure 1:**
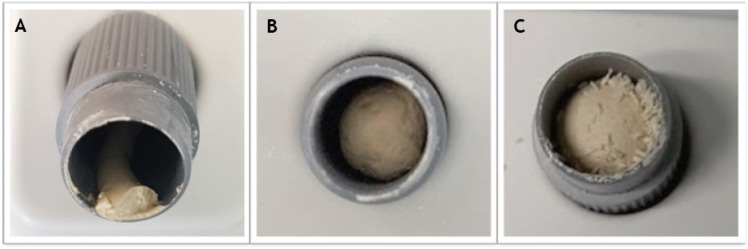
Mixing Biodentine with chitosan. (**A**) Commercial (unmodified Biodentine). (**B**) Biodentine + 5 wt% chitosan MMw. (**C**) Biodentine + 10 wt% chitosan MMw. Notably, the cement paste became stiffer when Biodentine was mixed with chitosan powder.

Commercially available Biodentine™ [BD (Septodont, Saint-Maur-des-fossés, Cedex, France)] is a powder and liquid system available in pre-set capsules with 700 mg of powder, in which five drops of Biodentine liquid are to be added [5] (Table 1). In this study, Biodentine powder was mixed with four different proportions of chitosan medium molecular weight powder [CS-MMw (Sigma-Aldrich, St. Louis, MO, USA)]: 2.5 wt%, 5 wt%, 10 wt%, and 20 wt%. To assure consistency between samples, Biodentine powder in each capsule was reweighed to an exact 0.7 g and then mixed with 180 µL of the manufacturer’s liquid (five drops of Biodentine liquid = approximately 180 µL). Incorporating higher concentrations of chitosan (>2.5 wt%) needed higher volumes of Biodentine liquid to triturate the new mixture, as shown (Supplementary Table S1). The resultant powder was mixed with the manufacturer’s Biodentine™ liquid component in a mixing machine at 4000–4200 rpm for 30 s (Figure 1). After mixing, Biodentine was compacted into assigned molds, and each specimen was then kept at 37 °C in a humid atmosphere (around 95–100% relative humidity) for a specified time. Modified specification tests of the International Organization for Standardization (ISO) 6876 [26] were adapted for material tests, unless otherwise stated. All specimens were mixed by one operator.

### 2.1. Setting Time 

Biodentine samples (n = 7) were mixed and compacted into a stainless-steel ring mold, having a height of 2 mm and an internal diameter of 13 mm. Prior to use, the molds were kept in a humid cabinet at 37 °C. Each ring mold was placed on a glass plate and filled with the allocated material group, and the excess material was then removed to obtain a flat surface. The assembly was stored in a moist atmosphere in the cabinet at 37 °C for approximately 9 min. The test was performed using a Vicat apparatus E055N (Matest, Caerphilly, UK), consisting of a sliding rod weighing 300 g, and a removable needle with a cylindrical tip and a flat end (1.13 mm in diameter). The needle was carefully lowered vertically onto the horizontal surface of the material without exerting further pressure. The final setting time was recorded as the time taken from the end of mixing to the time at which the needle failed to leave an indentation on the set cement surface. 

### 2.2. Solubility 

Test samples (n = 6) were prepared using ring metal molds with an internal diameter of 13 mm and a height of 2 mm. Prior to use the testing, all ring molds were individually weighted (W_0_). The ring molds were filled with the allocated material group on a glass slab, and excess material was removed. The specimens were left to set for 24 h in a humid atmosphere at 37 °C. All test specimens were then weighted individually in their molds before immersion in water (W_1_). Initial dry weight (IDW) was recorded as the differences found between W_1_ and W_0_. The samples were individually immersed in a clean glass tube containing a fresh aliquot of 20 mL of sterile distilled water. Each assembly was placed in such a way that both surfaces were freely accessible to the immersing solution without contacting the walls of the glass containers. The specimens were then transferred into the incubator at 37 °C, where they were stored for one day. The specimens were then removed, after 24 h, from the glass tubes and rinsed with 1 mL of distilled water collected into the same tubes to remove loose debris from decomposition. The samples were left for complete drying for 48 h at 37 °C and then reweighed with their molds (W_2_). Final wet weight (FWW) was recorded as the differences found between W_2_ and W_0_. All measurement readings were in grams and recorded to four decimal places, to the nearest 0.0001 g. The amount of solubility was calculated to the nearest 0.001% using the following equation: Solubility (%) = ([IDW − FWW]/IDW) × 100.

### 2.3. Radiopacity 

Cement specimens (n = 6) were prepared with a thickness of 1 mm and a diameter of 7 mm. Test samples were kept for 24 h in a humid incubator at 37 °C. Each specimen was digitally radiographed with an aluminum step wedge (aluminum purity at 96%, 30 mm long × 15 mm wide, having 5 steps with a thickness of 2.5, 3.5, 4.75, 7, and 9 mm). The digital X-ray machine (Gendex 765DC, Gendex, Des Plaines, IL, USA) is operating at 65 kV, a current of 7 mA, and an exposure time of 0.020 s. The ImageJ program (National Institutes of Health, Bethesda, MD, USA) was used for image analysis. The mean gray pixel values of various thicknesses of the aluminum stepwedge were obtained and plotted against their thickness. After calibration, the mean gray pixel value for each specimen was automatically expressed in mm of aluminum (mmAl). 

### 2.4. Compressive Strength 

**Figure 2:**
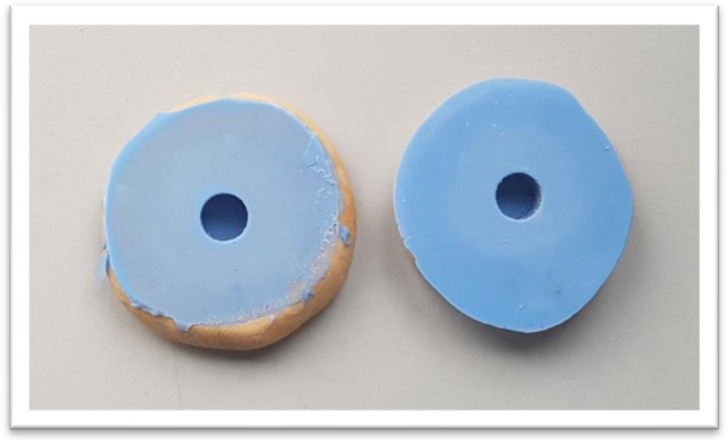
Molds (6 mm in diameter × 6 mm in height), corresponding to the size of metallic cylinders, were fabricated from dental silicone-based impression materials. The material was filled into each cylindrical hole with a slight excess. A glass slap was then placed on top of the filled molds and pressed down to remove the excess material.

A modified test of ISO 9917-1 [27] was adapted for testing the compressive strength (CSI). Cylindrical specimens (n = 10) were prepared with a height of 6 mm and a diameter of 6 mm using silicone molds, as shown in Figure 2. All cylinders were stored in a humid atmosphere at 37 °C for 30 days. Prior to the testing, the two circular faces of each cylindrical specimen were polished by a stainless-steel polishing strip to create two smooth, flat surfaces parallel to each other. The compressive strength for each Biodentine cylinder was measured using an Instron 3367 Universal Testing Machine [30 KN Static Load Cell (Instron, Buckinghamshire, UK)] at a speed of 1 mm/minute. Each material cylinder with its flat ends was placed between the platens of the apparatus. The load was applied in a direction parallel to the long axis of the cylinder until the samples were crushed. The maximum load required to break each test sample was recorded. The CSIs (σ_c_) were calculated in Megapascals using the formula: σ_c_ (MPa) $=\frac{4F}{\pi d^{2}}$ (N/mm^2^), where *F* is the maximum force applied (N), and *d* is the mean diameter of the specimen in mm. 

### 2.5. Microhardness 

A modified test of ISO 6507-1 [28] was adapted for Vickers microhardness (HV) test. Disc-shaped specimens of Biodentine (n = 5) were prepared by compacting the material into stainless-steel ring molds (13 mm in diameter and 2 mm in thickness) 30 days before the test. The specimens were kept in a moist atmosphere at 37 °C. The surface microhardness testing was performed using a Vickers microhardness tester (Krautkramer TIV, [GE Inspection Technologies, Coventry, UK]) with a Vicker diamond indenter point. Three indentations were made on one polished cement surface of each specimen. The image of each indentation was transferred and evaluated automatically. The mean Vickers hardness number (VHN) value was recorded for each. 

### 2.6. Surface Topography 

Biodentine microstructure was observed using scanning electron microscopy (SEM). Cement specimens were prepared (1 mm in thickness × 7 mm in diameter). The specimens were stored in the humid cabinet for a month before being imaged. Samples were then taken for gold/palladium sputter coating and mounting. Digital images were acquired using Jeol JSM-IT100 InTouch™ scanning electron microscope. Representative images of the samples were taken at a magnification of ×800.

### 2.7. Cell Proliferation 

Human embryonic kidney (HEK 293) cells [29] obtained from the American Type Culture Collection (ATCC CRL-1573 [Manassas, VA, USA]) were cultured and seeded in 75-cm^2^ flasks in Knock-out Dulbecco’s modified Eagle’s medium (DMEM-KO [Gibco, Loughborough, UK]) supplemented with 10% fetal bovine serum, L-Glutamine, and Penicillin-Streptomycin solution. Cells were passaged by detaching with trypsin–EDTA [0.25%, (Gibco™, Loughborough, UK)] when flasks were approximately 90% confluency. Cultures were maintained at sub-confluent levels in a humidified atmosphere at 37 °C with 5% CO_2_. Biodentine ± CS-MMw discs (2 mm in thickness × 7 mm in diameter) were prepared and kept in the humid atmosphere for 24 h. Material specimens were disinfected for 30 min by UV. The samples were then placed in a 24-well plate, one disc per well, in 1 mL DMEM-KO and kept overnight inside a 5% CO_2_ incubator at 37 °C. HEK 293 cells were plated into a 96-well plate at a seeding density of 10^4^ in DMEM-KO and allowed to adhere overnight. The following day, the cell’s medium was replaced with the material leachate. To observe a dose-response relationship, the material extracts were serially diluted with DMEM-KO, as previously described by Zhou and Shen [30]. This was to achieve a total of three concentrations of each extract. To evaluate the effect of chitosan on proliferation, cell metabolism was assessed after 72 h of incubation, using a colorimetric MTT assay (Sigma-Aldrich, Gillingham, UK). Following incubation, and discarding, MTT solution was added into each well and incubated for 4 h at 37 °C and 5% CO_2_. Subsequently, the solution was replaced with dimethyl sulfoxide (DMSO [Sigma-Aldrich, Gillingham, UK]) and further incubated for 1 h at 37 °C and 5% CO_2_. Absorbance readings were performed on a microplate reader (FluoStar Omega, BMG Labtech, Aylesbury, UK) at 545 nm wavelength and 630 nm as a reference wavelength. Three biological repeats were carried out for each condition in triplicate. 

### 2.8. Statistical Analysis 

All graphs, data distribution, and statistical analysis were performed with GraphPad Prism version 9 (GraphPad, San Diego, CA, USA). Data distributions were assessed, before analysis, using the D’Agostino-Pearson omnibus normality test (samples ≥ 8) and the Shapiro–Wilk normality test (samples < 8). Kruskal–Wallis and Dunn’s tests were used to determine the *p* values for multiple comparisons of non-parametric data. An ANOVA test with either Tukey’s or Dunnett’s tests was used for multiple comparisons of normally distributed parametric data. Differences were considered statistically significant when *p* < 0.05.

## 3. Results

### 3.1. Setting Time 

**Figure 3:**
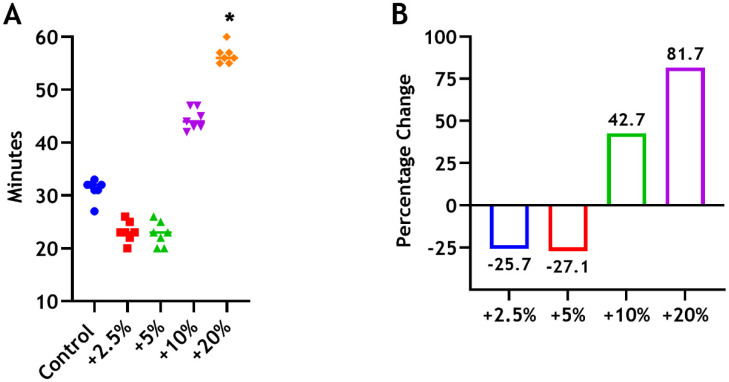
(**A**) Setting time of Biodentine material following the incorporation of 2.5 wt% (red), 5 wt% (green), 10 wt% (purple), and 20 wt% (orange) of CS-MMw. The control (blue). (**B**) The percentages of reduction and increase in the setting time compared with the control (unmodified) group.

The final setting time of Biodentine was determined to be 31.14 (±1.95 min). Slight decreases in setting time by 25.7% and 27.1% were observed following the inclusion of 2.5 wt% and 5 wt% chitosan, respectively. In contrast, the mean setting times for Biodentine incorporated with 10 wt% and 20 wt% CS increased by 42.7% and 81.7%, respectively, compared with the commercial Biodentine material (Figure 3). The difference between the control and the 20 wt% group was statistically significant (* *p* < 0.05). 

### 3.2. Solubility

**Figure 4:**
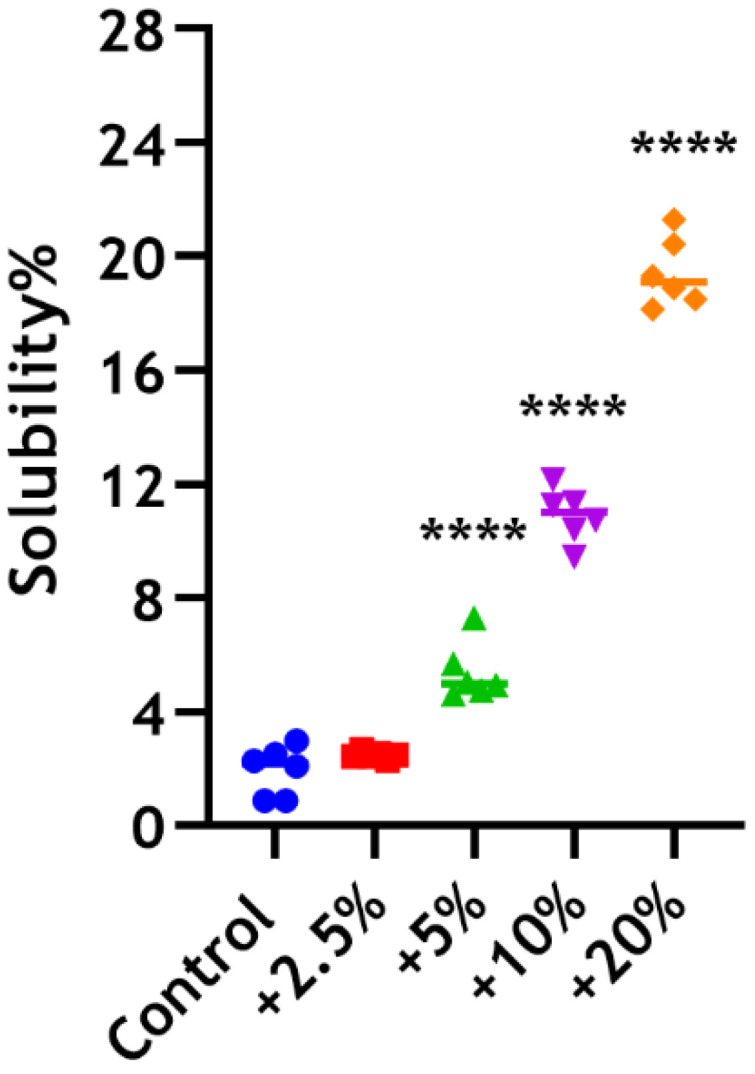
Solubility percentages of BD ± CS following the incorporation of 2.5 wt% (red), 5 wt% (green), 10 wt% (purple), and 20 wt% (orange) of CS-MMw, after immersion in sterile distilled water for 24 h. The control unmodified group (blue).

The unmodified control and Biodentine material incorporated with 2.5 wt% chitosan showed a low solubility of 1.94% and 2.47%, respectively, following 24 h of immersion in sterile water, while the weight loss of Biodentine incorporated with 5 wt% was 5.38%. In contrast, the test groups of the material with 10 wt% and 20 wt% CS provided significantly higher solubility than the control at approximately 11% and 19% (**** *p* < 0.0001), respectively (Figure 4). 

### 3.3. Radiopacity 

**Figure 5:**
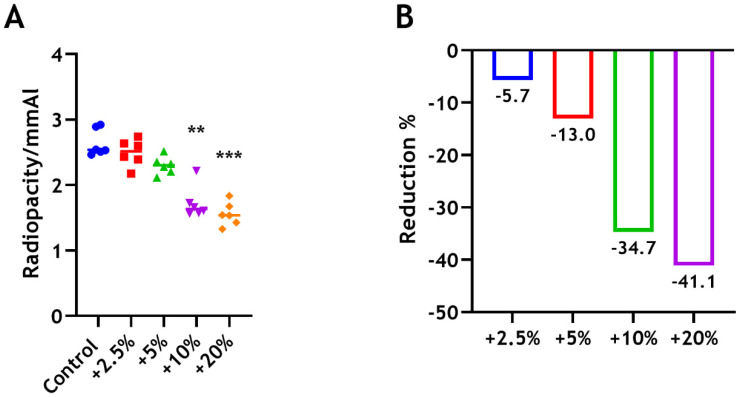
(**A**) Radiopacity of BD ± CS following the incorporation of 2.5 wt% (red), 5 wt% (green), 10 wt% (purple), and 20 wt% (orange) of CS-MMw. The control unmodified group (blue). (**B**) The percentages of reduction in radiopacity compared with the control (unmodified Biodentine) group.

Based on the results shown, the radiopacity of the unmodified material was 2.64 (±0.21 mm Al). The radiopacity of BD blended with 2.5 wt% and 5 wt% CS decreased, but not significantly, by 5.7% and 13%, respectively, compared with unmodified material. The 10% and 20% groups were significantly less radiopaque than the control group (** *p* < 0.01, *** *p* < 0.001), with approximately 35% and 41% reductions, respectively (Figure 5). 

### 3.4. Compressive Strength 

**Figure 6:**
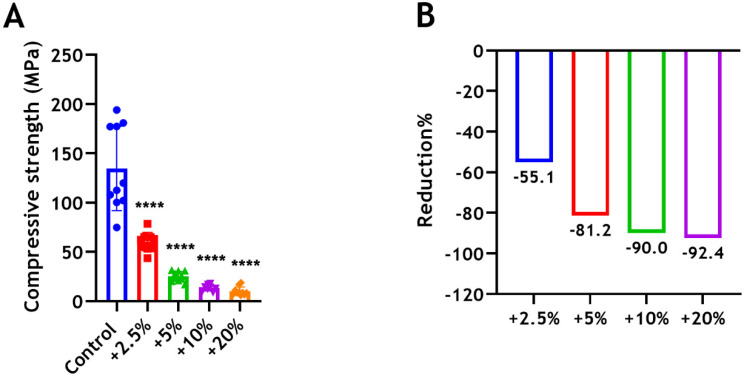
(**A**) Compressive strength of BD ± CS-MMw following the incorporation of 2.5 wt% (red), 5 wt% (green), 10 wt% (purple), and 20 wt% (orange) of CS-MMw. The control unmodified group (blue). Two defective specimens were excluded. (**B**) The percentages of reduction in CSIs compared with the control.

The addition of CS significantly reduced the compressive strength of BD in all groups in a dose-dependent manner (**** *p* < 0.0001). Unmodified Biodentine showed a significant superior strength with a value of 134.78 MPa. Adding 2.5 wt%, 5 wt%, 10 wt%, and 20 wt% CS-MMw, decreased the strength by 55%, 81%, 90%, and 92%, respectively, compared with the unmodified control (Figure 6). Significant changes were also found between the 2.5% and 5% groups (*p* < 0.01). However, no significant changes were seen between the 5% and 20% groups (*p* > 0.05).

### 3.5. Microhardness

**Figure 7:**
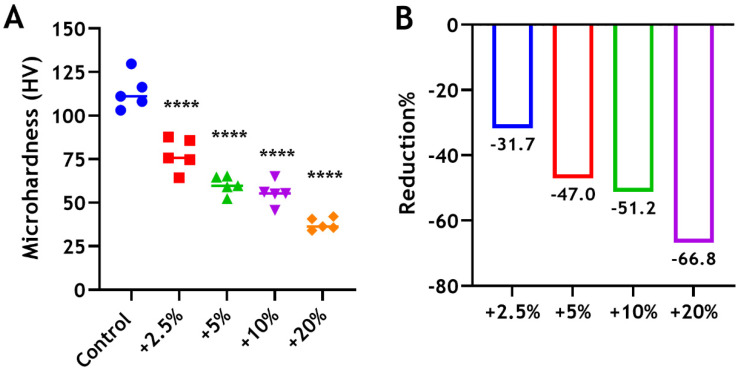
(**A**) Microhardness of BD ± CS following the incorporation of 2.5 wt% (red), 5 wt% (green), 10 wt% (purple), and 20 wt% (orange) of CS-MMw. The control unmodified group (blue). (**B**) The percentages of reduction in microhardness compared with the control.

The mean Vickers microhardness for the unmodified BD was 113.6 HV. Adding 2.5 wt%, 5 wt%, 10 wt%, and 20 wt% CS-MMw to Biodentine was found to reduce the microhardness (MH) of the material significantly (**** *p* < 0.0001) by approximately 32%, 47%, 51%, and 67%, respectively (Figure 7). ANOVA with Tukey’s tests showed significant differences between the 2.5% and 5% groups (*p* < 0.05), and between the 5% and 20% groups (*p* < 0.01). The mean values of the tested groups are summarized below (Table 2). 

### 3.6. Cell Proliferation 

**Figure 8:**
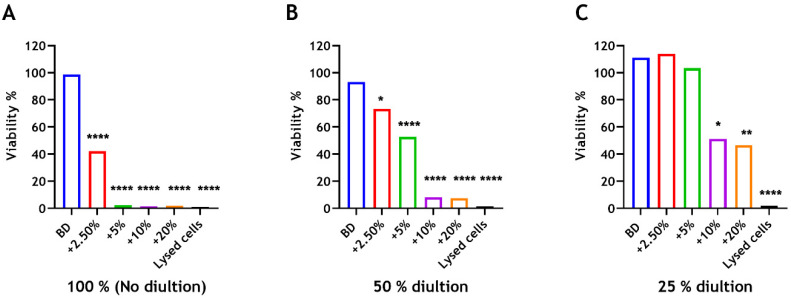
The cell viability% of extracts derived from BD ± CS with various concentrations: (**A**) No dilution. (**B**) 50% dilution; (**C**) 25% dilution, following cell culture for 72 h. Chitosan concentrations: 2.5 wt% (red), 5 wt% (green), 10 wt% (purple), and 20 wt% (orange). The unmodified Biodentine material (blue). Cell viability was calculated relative to the positive control. Three parallel experiments were performed in triplicate (n = 3).

Results of the viability assay showed high cell viability of Biodentine-treated cells at all extract concentrations, with no significant differences compared with the positive control (cells cultured without material extracts). The undiluted extract (100%) from Biodentine presented viable cells at 98.5%. In contrast, cells cultured with undiluted material extracts derived from BD-CS groups at all CS concentrations showed significant reductions in viability compared with the control group (**** *p* < 0.0001). In the meantime, the viability for the 2.5% group was significantly higher than the other BD-CS groups (**** *p* < 0.0001). Cell viability of BD-chitosan extract at 2.5 wt% was 42.2%, whereas cells exposed to undiluted BD-CS extracts at 5 wt%, 10 wt%, and 20 wt% displayed the lowest viabilities at 2.4%, 1.5%, and 1.8%, respectively (Figure 8A). With 50% diluted extracts, higher percentages of cells viabilities cultured with BD-chitosan were observed (Figure 8B) but were significantly less viable than the control (* *p* < 0.05 at 2.5 wt% and **** *p* < 0.0001 at 5 wt%, 10 wt%, and 20 wt%). However, significant decreases in viability were only observed with 10 wt% (* *p* < 0.05) and 20 wt% (** *p* < 0.01) at 25% diluted extracts (Figure 8C).

### 3.7. Surface Topography 

**Figure 9:**
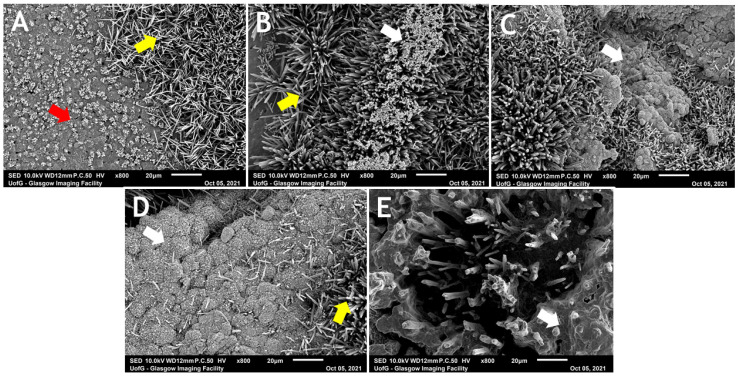
Scanning electron microscopy of BD discs. (**A**) Unmodified BD (control). (**B**) BD + 2.5 wt% CS. (**C**) BD + 5 wt% CS. (**D**) BD + 10 wt%. (**E**) BD + 20 wt%. SEM images show clumps of chitosan (white arrows). Yellow and red arrows indicate needle-like structures and intergrowth structures, respectively. Representative images were taken at a magnification of ×800.

The surface microstructure of the Biodentine shows superficial, small intergrowth structures, and needle-shaped crystals (Figure 9A). In the material-chitosan groups, small particles of chitosan at 2.5 wt% are observed dispersed within the needle-like structures (Figure 9B). From 5 wt% and onwards, clusters or larger aggregations of chitosan are seen. The modified form of the cement with a larger amount of chitosan microparticles showed fewer crystalline structures compared with the commercial Biodentine (Figure 9C–E).

## 4. Discussion

The long time of setting is seen as one of the most problematic features of calcium silicate-based materials. These prolonged setting times cause difficulty in material placement, where a material will not maintain its shape and is susceptible to being washed out before it is fully set [31]. Biodentine has a reduced setting time when compared with other calcium silicate-based materials, such as mineral trioxide aggregate [32]. This is perceived as one of its main clinical advantages [33]. In the literature, measurement of setting time has been performed using various methods. The main principle of testing remains based on the resistance of a needle to penetrate the cement surface, either when the needle fails to make a trace on the surface of the material [26] or when it fails to create a complete circular indentation in the cement [27]. According to the manufacturer, the final setting time was determined at around 10–12 min, when the elastic modulus of the material reached 100 MPa [5]. However, the final setting of Biodentine, in our study, was approximately three times longer than that described by the manufacturer. This increase in setting time was observed before by other researchers. Grech, Mallia [34], Elsaka, Elnaghy [35], and Kaup, Schäfer [36] evaluated the final setting time of BD to be 45 min, 44 min, and 85.66 min, respectively, when an indentation on the material surface was no longer visible. The differences between these results are probably attributable to slightly different experimental methodologies. However, the exact value for the baseline material is probably less important than the effect of the additions to the recorded setting time. In this study, the results showed some decreases in the setting time when small amounts of CS-MMw (2.5 wt% and 5 wt%) were incorporated. From 10% onwards, there was a retardation in the setting time. A creamy, putty-like consistency of the unset cement was obtained after mixing the unmodified Biodentine. However, less plastic material was obtained, possibly equating to a reduced water-to-powder ratio during mixing when 2.5 wt% and 5 wt% CS-MMw were added. It has been shown that an increase in the water-to-powder ratio increased Biodentine’s setting time [37]. Thus, reductions in set times could have been achieved by decreasing the amount of the free mixing Biodentine liquid, which subsequently led to setting acceleration. Paradoxically, increasing chitosan content up to 10 wt% and 20 wt% produced a grainy, crumbly material and retarded the setting reactions. Biodentine has been shown to set through a hydration reaction [38]. The addition of larger amounts of chitosan seemed to affect the hydration reaction of the material adversely by interrupting the connectivity between the cement grains, which results in an extended setting time.

One of the most important properties determining the durability of dental materials is their resistance against dissolution or disintegration [39]. The percentage loss in mass of the BD samples was recorded by measuring the decrease of the test specimens after storage in distilled water, as described in other studies [36,40]. The specimens were left to dry for 48 h to avoid an underestimation of weight loss due to water uptake. The unmodified cement and the 2.5 wt% CS groups attained the ISO standard by showing a weight loss of less than 3%. Other authors reported a solubility of <3% for commercial BD after 24 h [36]. The chitosan groups from 5 wt% and onwards have shown higher disintegration. This may have been attributed to the reduction in the connectivity of the material network structure by increasing the gaps between the particles of the cement when a larger amount of chitosan was blended into the cement, where a chemical bond between the chitosan additives and the material was not evident. It is, however, unclear whether the high solubility recorded in ‘in vitro’ studies has an adverse effect clinically [41]. It has been reported in a systematic review and meta-analysis that calcium silicate-based sealers have shown “worrying results”, for clinical use, in terms of solubility [41]. Nevertheless, the overall clinical success rate of endodontic treatment using calcium silicate sealer was over 90% [42]. 

Radiopaque materials are recommended for filling bone defects and root canals, to better define the filling quality and levels of resorption [43]. The radiopacity of 1 mm of dentine was shown to be equivalent to that of an equal thickness of aluminum, while enamel was twice as radiopaque as aluminum [44,45]. Accordingly, the ISO standard [26] established a minimum radiopacity corresponding to 3 mmAl for root canal (endodontic) sealing materials in clinical use. In our study, the radiopacity of Biodentine was found to be 2.64 mm Al. This is different from the manufacturer’s claims that the material possesses a radiopacity of 3.5 mm AI, complying with the ISO standard. Our results were in agreement with a number of other studies [46,47,48,49,50], which reported similar radiopacity values for Biodentine. According to Camilleri and Sorrentino [51], 5.1 wt% of zirconium oxide has been detected as a radiopacifier in Biodentine powder to facilitate detection on radiographs. However, inadequate contrast between Biodentine and surrounding structures was reported when assessed radiographically, which compromises the radiographic assessment of outcomes and follow-up [52]. Chitosan is organic and radiolucent. It is therefore not surprising that incorporation of CS into BD results in a reduction in radiopacity. However, the incorporation of 2.5 wt% chitosan has slightly reduced the radiopacity by approximately 6%. An endodontic material with low radiopacity is a significant clinical problem, resulting in a lack of clarity, which could lead to difficulties in the diagnosis and assessment of the treatment outcomes. 

While the compressive strength of Biodentine is not of primary concern, when placed as a retrograde filling or used for perforation repair, where the material does not bear any direct pressure. The compressive strength is relevant when used for direct pulp capping, where Biodentine must be able to withstand masticatory forces. In this study, we measured our samples at 30 days because it has been shown, in a previous study, that the compressive strength of the cement increased over time until reaching a maximum strength after one month, when compared with 1 and 7 days [53]. In our study, the compressive strength and microhardness of Biodentine have been determined to be 134.78 MPa and 113.60 HV, respectively. To explain the matter further, Biodentine less porous than other tricalcium silicate materials [54]. The reduction of the manufacturer’s water quantity due to the presence of the hydrosoluble polymer “water reducing agent” [5] reduces the porosity of Biodentine. It has been known that Biodentine sets through a hydration process that produces calcium silicate hydrate (CSH) and calcium hydroxide [Ca(OH)_2_] [38]. The material porosity is mainly filled by C-S-H and by Ca(OH)_2_, to a lesser extent [51]. Biodentine, therefore, gains strength and becomes denser through the hydration reaction. The reductions in both microhardness and compressive strength shown by increasing the amount of chitosan incorporation occur in a dose-dependent manner. These observations suggest that the interface between the hydration products of Biodentine and chitosan particles was weak. The addition of chitosan seemed to disturb the hydration reaction and crystalline structure of Biodentine, which resulted in compromising the mechanical properties of the material greatly. According to Luković and Schlangen [55], the development and propagation of microcracks within a cement are controlled by the distribution of local stresses and weak links in the cement structure. The heterogeneity of the cement has a great impact on this process. High heterogeneity leads to microcracking almost through the whole specimen [55]. In fact, it has been shown that the interconnections and strong chemical bonding between the limestone filler and C-S-H result in strength development and a very high resistance to crack propagation after applying a mechanical load [56]. In our study, the compressive strength of the 2.5% group was approximately 61 MPa, which is comparable to those obtained for glass ionomer (47 MPa) [57], super ethoxybenzoic acid (60–78 MPa), intermediate restorative material (52–57 MPa), and MTA (40–67 MPa) [58]. However, compressive strength is not an important factor to consider when a filling material, such as root-end fillings and liners, does not bear a direct pressure. Thus, the clinical use of the modified Biodentine with a smaller amount of chitosan could be limited to those applications where the bioactivity of the material can be beneficial but a high compressive strength is not necessarily required. 

Scanning electron microscopic analysis of the surface of hydrated Biodentine revealed intergrowth structures accompanied by and covered by needle-shaped crystals. The former was thought to be a form of C-S-H, one of the main products of the hydration process of tricalcium cements [51]. The lack of superficial deposition of hexagonal plate-like crystals of Ca(OH)_2_ (Portlandite) is thought to be due to the low porosity of Biodentine which discourages the deposition and attachment of crystals [38]. In previous studies, needle-like structures on the Biodentine surface, stored in a humid atmosphere, was observed [59] when Biodentine was exposed to different acidic pH values [60]. These crystals are believed to be calcium carbonate (calcite) [33]. The chitosan microparticles have been seen in dispersion within the material, in clumps, particularly at 10 wt% and 20 wt% chitosan, which seemed to interfere with the crystalline formation. Highly disturbed crystalline structures could explain the significant reductions in the mechanical properties of Biodentine. In relation to this concept, the formation of needle-like crystals in MTA is believed to be significant in interlocking the entire material mass, and their absence can reduce the material hardness [61].

Materials used as a root-end filling, such as Biodentine, are placed in intimate contact with apical bone and periodontal tissues. It is therefore essential to ensure that they are biocompatible with the living host tissues [30]. Biocompatible materials may not be entirely "inert." A bioactive material is one that elicits a specific biological response in adjacent cells, which results in promoting the healing process [62]. In the literature, the biocompatibility tests of dental materials generally assess the cytotoxicity of a material relative to certain cell lines. In this study, the human embryonic kidney cell was selected as an in vitro model to assess cell viability. This cell line is widely used to evaluate cytotoxicity [63,64]. The MTT-based colorimetric assay was used to assess cell viability, which measures cellular metabolic activity as an indicator of cell viability and proliferation [65]. Based on the results, Biodentine does not affect cell proliferation, following 72 h of incubation, compared with the control (cells only). The high cell viability could be attributed to the constituents of the material itself, which are mainly composed of tricalcium silicate and dicalcium silicate [66]. The biocompatibility and bioactivity of Biodentine have been reported by other researchers [1,67,68]. The metabolic activity of cultured cells was significantly reduced with the addition of chitosan in a dose-dependent manner, although the cellular viability for the 2.5% group was significantly greater than the other Biodentine-chitosan groups. Notably, diluted extracts from modified BD showed higher percentages of cell viability than undiluted extracts, with a similar trend observed amongst all CS concentrations. This is relevant clinically, as when an endodontic filling material is placed as a retrograde or an orthograde filling in vivo, leached substances may be diluted by tissue fluids. Material extracts, therefore, were performed to simulate a possible dose-response effect occurring in vivo [69]. Reduced metabolic activity might be a precondition for cellular cytotoxicity. The cytotoxicity in human liver cells has been delineated by Loh and colleagues following exposure to higher concentrations of medium molecular weight chitosan nanoparticles (CSNPs), where cellular uptake of CSNPs into the nucleus at a nanoparticle concentration of 1% *w*/*v* was visualized, and the cell membrane integrity was compromised as evidenced by enzyme leakage [70]. Another study suggested that low concentration of CSNPs (10 and 100 µg/mL) were relatively nontoxic to mouse hematopoietic stem cells, and the cytotoxicity was concentration-dependent [71]. Although we have used the material leachate, these findings may indicate that the incorporation of chitosan in dental materials could be applicable in low concentrations, where the material’s antimicrobial properties can be beneficial against microbial biofilms.

Although powdered chitosan has been shown to enhance the antibiofilm efficacy of Biodentine [11], it could have greater potential applications in calcium silicate materials when used in low concentrations as a natural antimicrobial agent, as higher concentrations of the chitosan filler (>2.5 wt%) result in a diminution of material properties. In this study, we were limited to using insoluble chitosan due to financial implications and product availability. There may be merit in considering a repeat of this work with lower concentrations and different molecular weights of chitosan. 

## 5. Conclusions

The addition of chitosan MMw, in powder form, to Biodentine bioceramic has a predominantly adverse effect on the material properties. This effect is dose-dependent, thus, the material cannot be recommended. Optimization of chitosan integration into dental materials is necessary to help overcome these problems, and further work should be carried out regarding molecular weights, concentrations, and dilutions of chitosan.

## Figures and Tables

**Table 1:** Composition of Biodentine material, according to the manufacturer [5].

**Powder**	
Tricalcium silicate	Main core material
Dicalcium silicate	Second core material
Calcium carbonate and oxide	Filler
Iron oxide	Shade
Zirconium oxide	Radiopacifier
**Liquid**	
Calcium chloride	Accelerator
Hydrosoluble (water-soluble) polymer	Water reducing agent
Water	

**Table 2:** Mean values of the tested groups with standard deviations.

Test Group	Setting Time (n = 7)	Solubility (n = 6)	Radiopacity (n = 6)	Compressive Strength (n = 10)	Microhardness (n = 5)
BD Control	31.14 (±1.95 min)	1.94 (±0.87%)	2.64 (±0.21 mmAl)	134.78 (±42.84 MPa)	113.60 (±10.20 HV)
BD + 2.5 wt% CS	23.14 (±1.95 min)	2.47 (±0.13%)	2.49 (±0.20 mmAl)	60.56 (±9.43 MPa)	77.60 (±9.42 HV)
BD + 5 wt% CS	22.71 (±2.29 min)	5.38 (±1.01%)	2.30 (±0.14 mmAl)	25.27 (±5.05 MPa)	60.20 (±5.24 HV)
BD + 10 wt% CS	44.43 (±1.99 min)	10.88 (±0.93%)	1.73 (±0.25 mmAl)	13.48 (±3.60 MPa)	55.40 (±6.85 HV)
BD + 20 wt% CS	56.57 (±1.72 min)	19.42 (±1.21%)	1.56 (±0.18 mmAl)	10.20 (±4.34 MPa)	37.73 (±3.43 HV)

## Data Availability

Data is contained within the article.

## Supplementary Materials

The following supporting information can be downloaded at: https://www.mdpi.com/article/10.3390/dj12040100/s1, Table S1: Chitosan percentages (%) incorporated into Biodentine material.
