# Supplementary material for: The Effect of Chitosan Incorporation on Physico-Mechanical and Biological Characteristics of a Calcium Silicate Filling Material
Source: Dent J (Basel). 2024 Apr 10;12(4):100. doi: 10.3390/dj12040100 (PMC11048767; doi:10.3390/dj12040100)
Supplement: Supplementary file 1 [file dentistry-12-00100-s001.zip › dentistry-2811473-supplementary.pdf]

Supplementary Table S1. Chitosan percentages (%) incorporated into Biodentine material.

**Test group**	**BD-P (g)**	**CS-P (g)**	**BD-L (µL)**
BD Control	0.7	—	180
BD + 2.5 wt% CS	0.6825	0.0175	180
BD + 5 wt% CS	0.665	0.035	200
BD + 10 wt% CS	0.63	0.07	270
BD + 20 wt% CS	0.56	0.14	400

To assure consistency between samples, the dry powder of Biodentine was reweighed, before mixing, to exact 0.7 g as weight variations between Biodentine capsules were observed. The manufacturer liquid was measured and dispensed in microlitres. Biodentine powder weighing 0.7 g (BD-P) was mixed with 180 µL of the manufacturer Biodentine liquid (BD-L [5 drops of Biodentine liquid = 180 µL]). Incorporating higher concentrations of chitosan (> 2.5 wt%) required higher volumes of BD liquid to triturate the new composite.
